# The Long-Term Survival of LVAD Patients—A TriNetX Database Analysis

**DOI:** 10.3390/jcm13144096

**Published:** 2024-07-13

**Authors:** Nandini Nair, Balakrishnan Mahesh, Dongping Du

**Affiliations:** 1 Department of Medicine, Division of Cardiology, Penn State Health/PSUCOM, 500 University Drive, Hershey, PA 17033, USA; 2 Department of Surgery, Division of Cardiothoracic Surgery, Penn State Health/PSUCOM, 500 University Drive, Hershey, PA 17033, USA; bmahesh@pennstatehealth.psu.edu; 3 Department of Industrial, Manufacturing and Systems Engineering, Texas Tech University (TTU), Lubbock, TX 79409, USA; dongping.du@ttu.edu

**Keywords:** risk factors, LVAD long-term survival, TriNetX

## Abstract

**Background:** Donor shortage limits the utilization of heart transplantation, making it available for only a fraction of the patients on the transplant waiting list. Therefore, continuous-flow left ventricular assist devices (CF-LVADs) have evolved as a standard of care for end-stage heart failure. It is imperative therefore to investigate long-term survival in this population. **Methods:** This study assesses the impact of demographics, infections, comorbidities, types of cardiomyopathies, arrhythmias, and end-organ dysfunction on the long-term survival of LVAD recipients. The TriNetX database comprises de-identified patient information across healthcare organizations. The log-rank test assessed post-implant survival effects, while Cox regression was used in the univariate analysis to obtain the Hazard Ratio (HR). All analyses were conducted using the Python programming language and the lifelines library. **Results:** This study identified CMV, hepatitis A exposure, atrial fibrillation, paroxysmal ventricular tachycardia, ischemic cardiomyopathy, renal dysfunction, diabetes, COPD, mitral valve disease, and essential hypertension as risk factors that impact long-term survival. Interestingly, hypokalemia seems to have a protective effect and gender does not affect survival significantly. **Conclusions:** This is the first report of a detailed long-term survival assessment of the LVAD population using a decoded database.

## 1. Introduction

Heart transplantation remains the gold standard for end-stage heart failure (ESHF). However, donor shortage limits the utilization of this modality of treatment, making it available for only a fraction of the patients on the transplant waiting list. Continuous-flow left ventricular assist devices (CF-LVADs) have therefore risen in importance as a standard of care for end-stage heart failure. Short, mid, and long-term survival rates as well as quality of life have been found to improve with more technologically sophisticated machines [1,2,3,4,5,6]. Advancements in technology have led to patients remaining on left ventricular support for >5 years with acceptable quality of life. Durable mechanical circulatory support (MCS) technology improved following the development of continuous-flow pumps and its FDA approval as a bridge to transplantation in 2008 and then as destination therapy in 2010 [7,8]. Additionally, the creation of “The Interagency Registry of Mechanically Assisted Circulatory Support” (INTERMACS), sponsored by the National Heart, Lung and Blood Institute registry, paved the way to improve patient outcomes through research [8]. Further improvements occurred with advances in VAD technology, reducing device-related adverse events. The most sophisticated line of pumps approved by the FDA uses fully magnetically levitated technology. Data from clinical trials have shown a significant reduction in pump thrombosis in the CF centrifugal pumps compared to the earlier CF axial flow pumps that were first approved by the FDA [1,9]. With fewer adverse events, there has been a tremendous increase in LVAD implantations. Evaluating the impact of risk factors on long-term survival projections in patients supported on LVADs is therefore needed with the increasing utilization of this technology. Our study has used the TriNetX database to identify risk factors and their impact on the long-term survival of LVAD-supported patients.

## 2. Materials and Methods

The TriNetX dataset comprises de-identified demographic information, lab results, and diagnoses from 4705 patients who underwent LVAD implantation between 2002 and 2022 across 47 healthcare organizations. Globally, the TriNetX dataset has 74 healthcare organizations, but within the United States, it has 47 healthcare organizations. The healthcare organizations deposit electronic healthcare record data anonymously for further processing in the TriNetX database. This study is IRB-exempt as the TriNetX database is completely decoded. Lab results include 4771 Logical Observation Identifiers Names and Code (LOINC) codes which are more specific than Current Procedural Terminology (CPT) codes for tests, representing over 7 million instances collected from 80,227 encounters preceding implantation. The analysis was focused on the most recent lab results before implantation, with an average interval of 18.0 ± 30.9 days between lab result collection and LVAD insertion.

More than 300,000 instances of primary diagnoses before LVAD implantation were extracted. The diagnosis right before implantation was used, with an average interval of 25.3 ± 34.6 days between diagnosis and implantation.

The present study examined the impact of six demographic variables (age, gender, race, ethnicity, marital status, and regional location), 264 lab results, and 326 diagnoses on LVAD patient survival. Lab variables were selected by excluding those with over 90% missing values. The log-rank test was used to assess post-implant survival effects, while Cox regression was used in the univariate analysis to obtain the Hazard Ratio (HR). Patients with unknown lab results were removed before the univariate analysis.

Diagnosis variables were chosen by excluding those with fewer than 100 patients with positive diagnoses to mitigate bias. Each diagnosis underwent a log-rank test to compare survival between patients with positive and negative diagnoses, with a significance threshold set at *p* < 0.0010. Unknown diagnoses were excluded from the analysis. Additionally, univariate Cox regression analysis was conducted to quantify HRs for diagnoses significantly affecting post-implant survival.

The present study excluded patients with multiple LVAD implantations and those ≤18 years old at the time of implantation. Patients with recorded death times were classified as deceased, while those without were considered surviving at the time of data collection. Time to event was computed as the duration between LVAD implantation and death or data retrieval. Samples with missing values or data errors (e.g., implantation is after recorded death) were excluded. All analyses were conducted using Python 3.12.3, the lifelines library version 0.28.0, the icd10-cm library version 0.0.5, and the icd9cm library version 0.2.1.

## 3. Results

### 3.1. Study Cohort 

**Figure 1:**
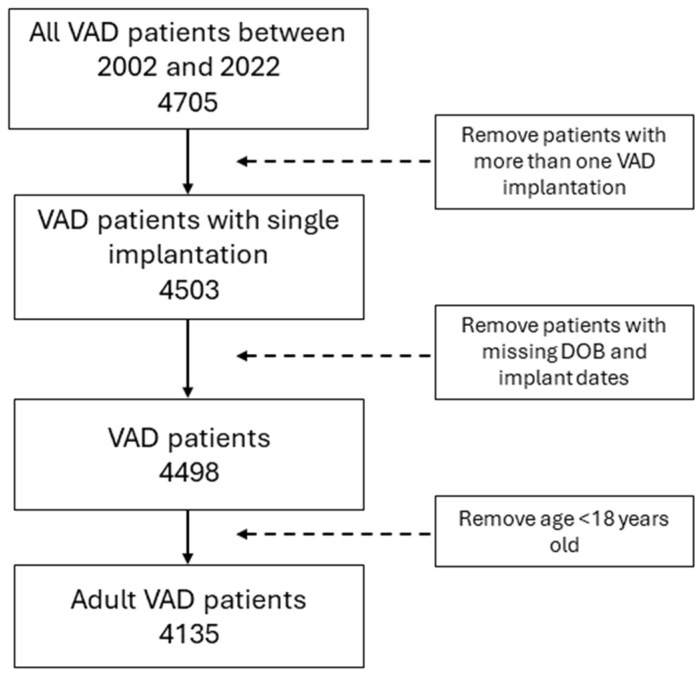
Derivation of study cohort.

The derivation of the study cohort is shown in Figure 1. All VAD patients who were implanted between 2002 and 2022 were included in this study. The final study cohort of 4135 patients was derived after excluding patients < 18 years of age, those who had more than one VAD implanted, and those with missing birth and implantation dates.

### 3.2. Demographics

**Figure 2:**
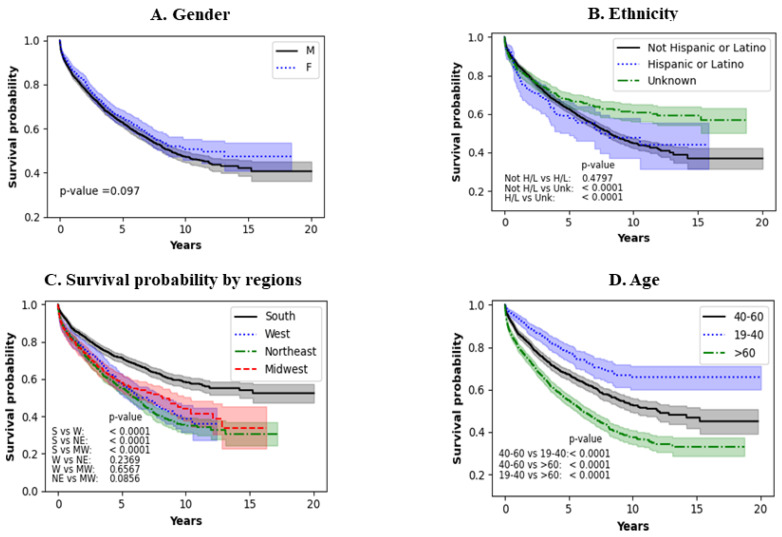
Impact of demographics on survival of LVAD patients.

Figure 2A shows the effect of gender. Males and females seem to have similar survival patterns (*p* = 0.097). Figure 2B shows the effect of ethnicity. When a detailed analysis was performed, non-Hispanic or Latino populations had no significant survival benefit compared to the Hispanic/Latino population (*p* = 0.4797). However, both Hispanic and non-Hispanic/Latino populations have a survival benefit when compared to the unknown group (*p* < 0.0001). This trend seems to extend throughout the analysis. Figure 2C shows survival probability by region. A detailed analysis shows that the South had a survival advantage versus the West, Northeast, and Midwest (*p* < 0.001). No survival benefit was noted when the West was compared to the Northeast and Midwest (*p* = 0.2369 and *p* = 0.8667, respectively). A comparison between the Northeast and the Midwest also shows no survival differences. Figure 2D shows the impact of age on LVAD survival. The worst survival was noted in patients >60 years of age and the best survival was noted in the younger population (19–40 years of age) throughout the period analyzed. Further analysis shows that when the 40–60 group were compared to the 19–40 group, the former had better survival (*p* < 0.0001), when the 40–60 group versus the >60 group were compared, the survival was better in the 40–60 group (*p* < 0.0001), and finally, when the 19–40 group versus the >60 group were compared, the 19–40 group had better survival (*p* < 0.0001).

### 3.3. Infections

**Figure 3:**
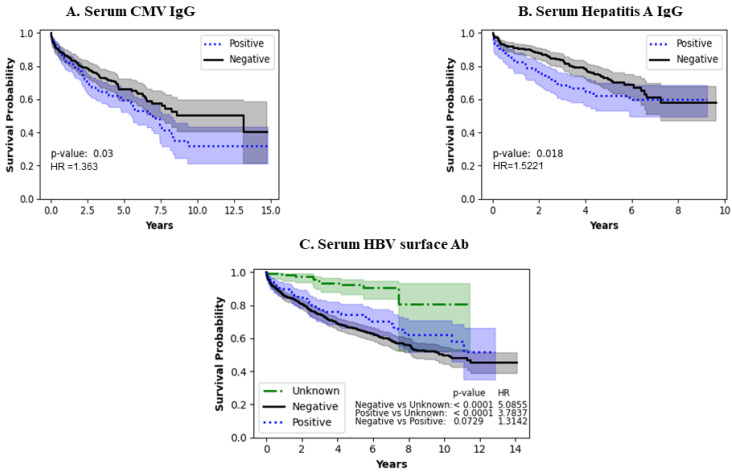
Impact of infections on survival of LVAD patients.

Figure 3A illustrates the impact of serum CMV IgG levels, showing better survival in the CMV-negative population and worse survival in the CMV IgG-positive population (*p* = 0.0300). Figure 3B shows the impact of hepatitis A viral infection, which shows populations with positive hepatitis IgG had worse survival close to approximately 8 years, after which the survival rate appears to be similar (*p* = 0.0180). Figure 3C shows the impact of the presence/absence of hepatitis B surface antibody on LVAD survival, with a trend of better survival in the hepatitis surface antibody-positive individuals (*p*-value < 0.0729). In addition, the hepatitis surface antibody-positive or -negative group, when compared to the unknown serology group, had a statistically significant survival benefit (*p* < 0.0001).

### 3.4. Comorbidities 

**Figure 4:**
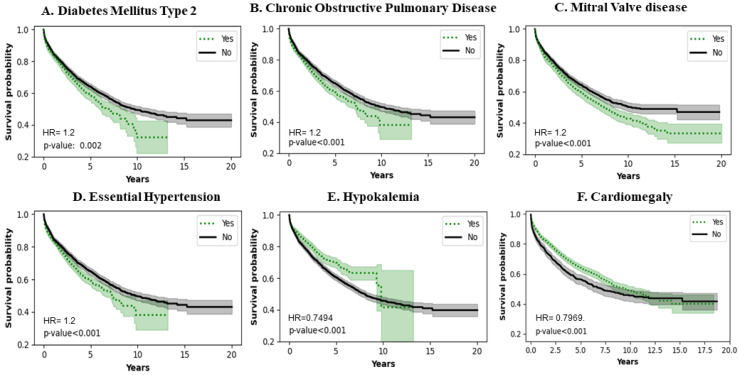
Impact of comorbidities on survival of LVAD patients.

Figure 4A shows the effect of Diabetes Mellitus type 2 (DM type 2). Patients with a history of DM type 2 have a poorer survival (*p* = 0.0020). Figure 4B shows the effect of Chronic Obstructive Pulmonary Disease (COPD). COPD patients have a lower survival probability (*p* < 0.0010). Figure 4C shows that mitral valve disease decreases survival probability (*p* < 0.0010). Figure 4D shows that essential hypertension also decreases survival probability in patients who carry the diagnosis (*p* < 0.0010). Figure 4E shows that persistent hypokalemia can increase survival probability up to approximately 10 years (*p* < 0.0010). Figure 4F shows that cardiomegaly has a protective effect on survival up to approximately 10 years (*p* < 0.0010).

### 3.5. Acute and Chronic Kidney Disease 

**Figure 5:**
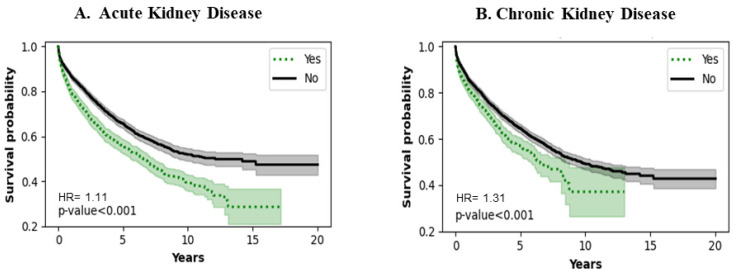
Impact of kidney disease on survival of LVAD patients.

Figure 5A,B show the effect of acute and chronic kidney disease, respectively. Patients with a history of acute and chronic kidney disease have poorer survival (*p* < 0.0010). The trend remains consistent throughout the entire period of analysis.

### 3.6. Type of Cardiomyopathy 

**Figure 6:**
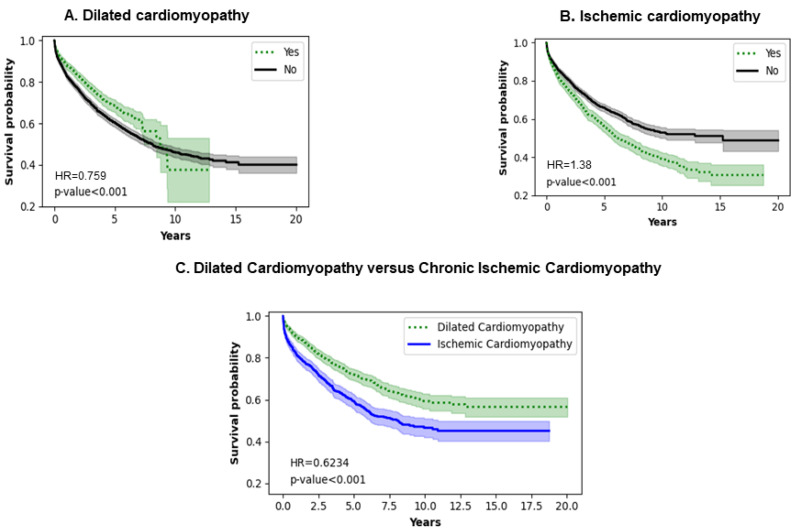
Impact of type of cardiomyopathy on survival of LVAD patients.

Figure 6A,B show the effect of dilated and ischemic cardiomyopathy, respectively. Patients with a history of ischemic cardiomyopathy have poorer survival (*p* < 0.0010). The trend remains consistent throughout the entire analysis. Interestingly, 50% of dilated cardiomyopathy patients are alive at 10 years and about 40% remain alive at 12 years. However, 50% of ischemic cardiomyopathy patients remain alive for close to 7.5 years, with a consistent decrease to 20% at 12 to 18 years. Figure 6C shows a comparison of dilated versus ischemic cardiomyopathy that shows that there is a statistically significant difference in the survival probabilities of the two types of cardiomyopathies, with dilated cardiomyopathy patients showing a higher survival probability throughout the entire period of analysis (*p* < 0.0010).

### 3.7. Impact of Type of Arrhythmias 

**Figure 7:**
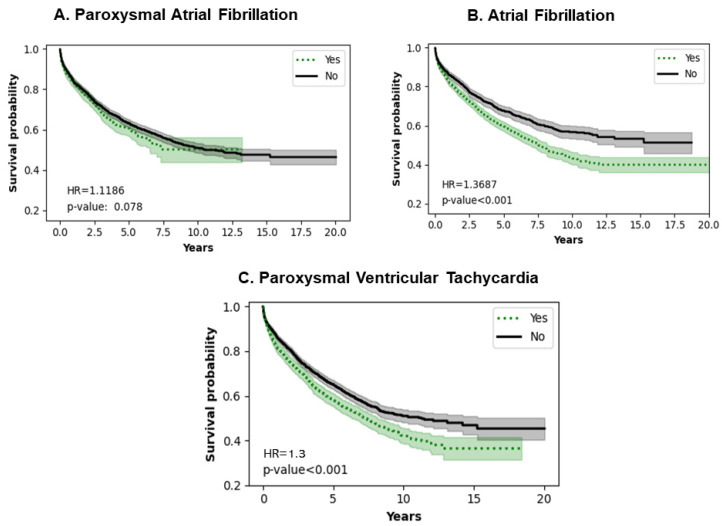
Impact of arrhythmias on survival of LVAD patients.

Figure 7A shows that paroxysmal atrial fibrillation does not affect the survival probability of LVADs in the long term (*p* = 0.0780). Figure 7B shows a statistically significant decrease in survival probability in atrial fibrillation (*p* < 0.0010). Figure 7C shows that paroxysmal ventricular tachycardia decreases survival probability (*p* < 0.0010).

Table 1 shows the baseline patient characteristics which include gender, race, ethnicity, regional location, and age.

All risk factors are summarized in Table 2. The number of positive and negative diagnoses is also depicted in this table.

## 4. Discussion

### 4.1. Demographics

The impact of gender is not statistically significant in our study throughout the duration of the analyses. Though women in general are fewer in number to receive VADs, their survival is not different in short-term studies conducted in a small number of patients up to 5 years [10]. This effect seems to be consistent with the results obtained in this study. Though perioperative differences in morbidity and mortality have been noted, the long-term effects do not seem to portray that trend. Though women supported on an LVAD are at a greater risk of neurologic events compared to their male counterparts, both men and women have similar all-cause mortality after any neurological events, thus implying no significant differences in survival [11]. In the current literature, decision-making and emotional factors affect women differently than men, which in turn can result in worse survival in the short term in women [12]. Further studies are needed to compare gender-based short and long-term quality of life in people and its impact on survival.

Our study shows no significant difference in survival between the non-Hispanic white race and the Hispanic race in the long term. The utilization of VADs has been assumed to be equal in all races when adjusted for other confounding factors in some studies [13]. Many other studies have shown that the utilization of VADS needs more diversity in terms of race, gender, and other socioeconomic issues. More studies are needed to assess race-based effects on long-term LVAD survival in the setting of improving diversity [14,15].

In our study, we have shown that the Northeast had the worst survival consistently throughout the analysis, while the South had the best survival. Studies using the INTERMACS data showed early on in 2015 that the South had lower survival compared to other regions [16]. However, such regional differences can be attributed to changes in the acuity of the patients and improvements in the utilization of bridging techniques to stabilize patients before implanting LVADs, which could have changed in centers contributing data to the TriNetX database from which we have extracted more current data. The data represented in the paper by Krim et al. were >10 years old, and hence, differences may be explained by changes in the utilization of technologically improved pumps in different centers in the various regions studied across the United States [16]. Improved survival in the South may also be due to changes in the demographic composition of these regions or underlying genetic or biological factors that confer a survival advantage to the population. It is possible that differences in data collection and sample size could have influenced our results. The TriNetX database has regional and global collaborative networks. The regional network in the US has 47 healthcare organizations that anonymously deposit data. Only the geographical location of these organizations was provided.

Long-term survival appears to be affected by age > 60 years, which is consistent with earlier findings that age > 60 is an independent risk factor for mortality in the short term at 1 year [17].

### 4.2. Infections

Different infections have varied effects on survival in the LVAD-supported population. CMV IgG antibodies, generated in response to CMV infection, do not appear to provide any long-term protective effect on survival in our study. It has also been shown in other studies that CMV reactivation occurs in the post-VAD implant period and could contribute to poor outcomes. CMV reactivation can occur in immunocompetent patients who are critically ill [14]. Impaired cellular immunity in LVAD patients leads to dysfunctional CD4-positive T cells, resulting in apoptosis until approximately 4 weeks post-implantation [18,19,20]. Our studies show that positive hepatitis A IgG patients have poorer survival for at least up to 8 years post-implant. This observation is difficult to explain as this is the first report, and more studies are needed to determine the implications. It is unclear if this has any link to impaired cell-mediated immunity in this population. The protection conferred by hepatitis B surface antibody can be explained by the fact that it indicates immunity acquired via an immune response to vaccination, or the presence of passively acquired antibodies or prior infection [21].

### 4.3. Comorbidities

Diabetes has been noted to be an important risk factor in our study for the long-term survival of LVAD patients. However, it has been noted that diabetes is not a short-term and intermediate risk factor but impacts long-term survival [22]. Such variations may be the effect of differential pre- and post-implant diabetes control. A meta-analysis showed that diabetes did not increase all-cause mortality in LVAD-supported patients [23]. COPD and LVAD patients appear to have lower survival in our study, at least up to 10 years. Other studies show no significant direct impact on survival in the short term but do have an impact on gastrointestinal bleeding, which is a risk factor for short-term survival [24,25]. Mitral valve disease in general seems to affect long-term survival in our study. This is consistent with earlier studies showing that greater than moderate mitral regurgitation is consistent with worse survival in the short and mid-term [26]. Most recently, a meta-analysis showed that post-operative mortality is not affected by moderate to severe MR [27]. However residual post-operative mitral regurgitation, though corrected to a certain extent, can impact prognosis via deterioration of right ventricular function and pulmonary pressures. The repair of mitral regurgitation may improve prognosis [28]. Additionally, residual mitral regurgitation may be influenced by differences in the gene expression of inflammatory markers which have been reported in ischemic mitral regurgitation [29].

Essential hypertension influences pump flows, causing them to decrease and thereby promoting stasis, which predisposes patients to pump thrombosis as well as strokes. Additionally, chronically low pulsatility leads to reduced endothelial function due to the absence of the routine cardiac cycle. Under such a milieu, small changes in blood pressure may deteriorate endothelial structure/function in the cerebral microvasculature and predispose the local area to progressive vessel damage and rupture, therefore also causing hemorrhagic strokes [30,31]. This could explain the significant impact of essential hypertension on the long-term LVAD survival seen in our study. Further studies are needed to elucidate this aspect of the physiological impact of LVADs. In a small study, hypokalemia showed a non-significant protective effect on the survival of LVAD patients 1-month post-LVAD implant [32]. In our study, hypokalemia seems to confer a survival benefit in the long-term. The effect of cardiomegaly on survival may be the end effect of molecular signaling pre- and post-LVAD implant, resulting in changes in myocyte size and a regression of hypertrophy. This phenomenon needs further investigation [33].

### 4.4. Renal Function

The long-term success and survival of LVAD patients are dependent on preserving end-organ function. Hence, it is not surprising that any insult to the kidneys, whether acute or chronic, has an impact on LVAD survival, as shown in our study. Additionally, LVAD support can mitigate or worsen renal function depending on the stage and acuity of heart failure, which impacts long-term survival [34,35].

### 4.5. Type of Cardiomyopathy

In our study, dilated cardiomyopathy patients did better than their ischemic counterparts in terms of long-term survival. The existing literature shows that in the short or long term, there is no significant decrease in mortality in ischemic cardiomyopathy patients [36,37]. Our findings can be explained by the higher burden of the comorbidities they may carry, such as older age, greater myocardial damage, and other structure/function issues that would impair the physiology of the heart and predispose them to more arrhythmias. In a small study, 12 dilated cardiomyopathy patients were explanted after fully functional recovery of the myocardium at 10 months post-implantation with no complications [38]. Larger studies are needed to compare the impact of the etiologies of heart failure on LVAD survival.

### 4.6. Arrhythmias

Our study showed that paroxysmal atrial fibrillation did not confer significant mortality on LVAD patients but atrial fibrillation in general had a significant effect on mortality in this population. Similar observations have been made in other studies in the short and mid-term [39,40,41,42]. Additional atrial fibrillation can also lead to ventricular tachyarrhythmias, which is an independent risk factor noted in our study [43]. Electrical storms impact short-term survival significantly [44].

The survival of LVAD patients can be impacted by several factors depending on the timing of LVAD implantation and the INTERMACS class. Additionally, HF patients have varied phenotypes which are highly heterogenous, contributing to differences in survival. Patients in cardiogenic shock have poor survival in the presence or absence of temporary mechanical circulatory support, while others who are in higher INTERMACS classes have a better 1-year survival. In this study, we have pooled all LVAD patients as the data were derived from a decoded database in which we did not assess INTERMACS classes. Estimating survival in patients with varied phenotypes is challenging. Hence, performing a survival analysis with large, decoded databases can show population averages but patient-specific risk stratification will vary widely. When predicting survival statistics based on risks, the accuracy depends on the homogeneity of the patient population studied. It also depends on using a risk prediction model that was derived from a patient population that resembles the study population. This investigation is unique in that we studied selected risk factors to assess their impact on patient survival in the long term.

## 5. Conclusions

This is the first report of a detailed long-term survival assessment of several risk factors that impact the LVAD population. Our study shows that older individuals, those of non-Hispanic/Latino race, and the Northeast populations had worse survival in terms of the demographics of the population. Exposure to CMV and hepatitis A does not confer a survival advantage but patients who had hepatitis surface antibodies had a significant survival advantage. Comorbidities such as diabetes, COPD, mitral valve disease, and essential hypertension showed worse survival. Hypokalemia and cardiomegaly conferred a survival advantage for up to 10 years in our study. Both acute and chronic kidney diseases significantly worsen long-term survival. In our study, dilated cardiomyopathy patients consistently showed better survival in the long term with LVAD. In our study, paroxysmal ventricular tachycardia and persistent atrial fibrillation patients showed poor long-term survival but there was no significant change in survival in the paroxysmal atrial fibrillation population.

### Limitations

This study is limited by the fact that it is retrospective and uses diagnosis codes instead of actual patient encounters. Large decoded publicly available databases are hampered by missing and incomplete data, which may preclude better risk prediction. This study also used the LVAD population as a whole because of the lack of avenues to classify patients according to INTERMACS classification which takes into account the acuity of the condition of the patients. It is also possible that differences in data collection and sample size could have influenced our results. The TriNetX database has regional and global collaborative networks comprising healthcare organizations that anonymously deposit data. Only the geographical locations of these organizations are available. This makes it impossible to gain any insight into the data’s quality.

Future directions would include internal and external validation of risk factors in different databases to evolve a risk factor assessment model for this population. Future studies are also needed to classify risk factors as short, mid, or long-term. The use of AI-driven technologies may reveal risk factors which may help increase the sensitivity and specificity of the risk factor models to better predict the survival of LVAD patients. Additionally, patient phenotypes and functionality should be factored into survival predictions. The use of homogenous study populations with fewer variations in phenotypes will help us to develop more accurate risk identification and prediction models.

## Figures and Tables

**Table 1:** Baseline patient characteristics.

	Number	%	Survived (%)	Deceased (%)	Years Survived (Mean ± SD)	*p* Value
**Gender**						0.0968
Male	3218	77.82	1246 (39)	1972 (61)	2.51 ± 2.59	
Female	917	22.18	323 (35)	594 (65)	2.46 ± 2.39	
**Race**						0.0005 *
White	2624	69.81	1070 (41)	1554 (59)	2.51 ± 2.54	
Black or African American	1064	28.31	338 (32)	726 (68)	2.57 ± 2.47	
Asian	37	0.98	10 (27)	27 (73)	2.04 ± 1.59	
American Indian or Alaska Native	27	0.72	10 (37)	17 (63)	3.41 ± 2.61	
Native Hawaiian or Other Pacific Islander	7	0.19	1 (14)	6 (86)	0.43 ± 0	
Unknown	376	10	140 (37)	236 (63)	2.23 ± 2.83	
**Ethnicity**						0.0008 *
Not Hispanic or Latino	3232	85.98	1270 (39)	1962 (61)	2.63 ± 2.57	
Hispanic or Latino	164	4.36	65 (40)	99 (60)	2.06 ± 2.28	
Unknown	739	19.66	234 (32)	505 (68)	1.91 ± 2.39	
**Regional Location**						<0.0001 *
South	1906	50.7	585 (31)	1321 (69)	2.67 ± 2.71	
Northeast	1341	35.67	590 (44)	751 (56)	2.34 ± 2.37	
Midwest	503	13.38	225 (45)	278 (55)	2.36 ± 2.55	
West	385	10.24	169 (44)	216 (56)	2.63 ± 2.54	
**Age**						<0.0001 *
>60	1802	47.94	827 (46)	975 (54)	2.35 ± 2.49	
40–60	1764	46.93	612 (35)	1152 (65)	2.62 ± 2.66	
19–40	569	15.14	130 (23)	439 (77)	2.82 ± 2.31	

* *p* < 0.0010.

**Table 2:** Risk factor assessment.

Code	Risk Factor	Number Positive	Number Negative	*p*-Value	Hazard Ratio
13949-3	Serum CMV IgG	268	286	0.0310 *	1.3630
32018-4	Serum hepatitis A IgG	159	364	0.0184 *	1.5221
22322-2	Serum HBV surface Ab	188	785	0.0010 *	0.7609
E11.9	Diabetes	2985	1037	0.0020 *	1.2001
416.8	Chronic Obstructive Pulmonary Disease	3018	1004	0.0010 *	1.2064
424	Mitral valve disease	3061	961	0.0010 *	1.2114
I10	Essential hypertension	2442	1580	<0.0010 *	1.2077
E87.6	Hypokalemia	2854	1168	<0.0010 *	0.7494
I51.7/429.3	Cardiomegaly	2522	1500	<0.0010 *	0.7969
N17.9	Acute kidney disease	2191	1831	<0.0401 *	1.1174
N18.9	Chronic kidney disease	3060	962	<0.0010 *	1.3100
I142.0	Dilated cardiomyopathy	2685	1337	<0.0010 *	0.7590
I25.5	Ischemic cardiomyopathy	2689	1333	<0.0010 *	1.3800
427.1	Paroxysmal ventricular tachycardia	2779	1243	<0.0010 *	1.300
427.31	Atrial fibrillation	2075	1624	<0.0010 *	1.3247
I48	Paroxysmal atrial fibrillation	980	2778	0.0780	1.1186
272.4	Hyperlipidemia	2761	1261	<0.0010 *	1.500
414	Coronary atherosclerosis of native vessel or graft	3069	953	<0.0010 *	1.500

* *p* < 0.0010.

## Data Availability

The data will be made available on request.
